# Robotic-assisted laparoscopic ureterocalicostomy (RALUC): a systematic review of its applications

**DOI:** 10.1007/s00345-025-06046-w

**Published:** 2025-12-17

**Authors:** Theodoros Spinos, Panagiotis Kallidonis, Vasileios Tatanis, Kristiana Gkeka, Angelis Peteinaris, Anja Dietel, Ho Thi Phuc, Doreen Trebst, Stefan Siemer, Toni Franz, Evangelos Liatsikos, Jens-Uwe Stolzenburg

**Affiliations:** 1 Department of Urology, University of Patras, Patras, Greece; 2 Department of Urology, University of Leipzig, Leipzig, Germany; 3 Department of Urology, University of Saarland, Homburg, Germany; 4 Department of Urology, Medical University of Vienna, Vienna, Austria

**Keywords:** RALUC, Robotic-assisted surgery, Reconstructive urology, UPJO, Minimally-invasive surgery

## Abstract

**Purpose:**

Ureterocalicostomy refers to the anastomosis between the lower pole calyces and the ureter. Robotic-assisted laparoscopic ureterocalicostomy (RALUC) is gaining ground, ultimately. The current systematic review summarizes all applications of RALUC in both adults and children.

**Methods:**

In line with the Preferred Reporting Items for Systematic Reviews and Meta-Analyses (PRISMA) Guidelines, three databases (PubMed, Scopus and Cochrane) were screened, from their inception to 16 February 2025. The following search string was used: robotic AND (ureterocalicostomy OR ureterocalicostomies OR ureterocalycostomy OR ureterocalycostomies).

**Results:**

Eight studies fulfilled all inclusion criteria and were finally considered for qualitative synthesis. The rate of patients who had undergone previous pyeloplasty ranged in included studies from 20% to 100%, while the rate of patients who had undergone a nephrostomy tube placement before the ureterocalicostomy ranged from 38% to 100%. Total operative time ranged from 157.6 (90–240) to 272 min, while estimated blood loss ranged from 27.5 (10–75) to 115 (50–200) mL. Reoperation rates ranged from 0% to 50%, while the success rates ranged from 66.7% to 100%. Finally, taking into consideration the Clavien-Dindo Classification System, the Grade I-II complications ranged from 0% to 40%, while the Grade III-IV ones ranged from 0% to 20%.

**Conclusion:**

RALUC is a feasible, safe and efficient procedure for patients with complicated ureteropelvic junction obstruction. The implementation of higher-quality studies on larger samples, including comparative ones and randomized controlled trials, is crucial in order to draw safer conclusions.

**Supplementary Information:**

The online version contains supplementary material available at 10.1007/s00345-025-06046-w.

## Introduction

Ureterocalicostomy refers to the anastomosis between the lower pole calyces and the ureter after the excision of the parenchyma of the lower pole of the hydronephrotic kidney. It is considered a primary or alternative treatment option after failed reconstructive procedures with the aim of restoring upper urinary tract continuity [1]. The indications of ureterocalicostomy comprise congenital ureteropelvic junction obstruction (UPJO), UPJO following complicated renal anatomical situations (horseshoe kidney, ectopic pelvic kidney, renal malrotation) and UPJO after failed pyeloplasty or repeated pyelolithotomies [1–8]. Other indications include long upper ureteral traumatic rupture, long upper ureteral strictures (greater than 4 cm), post-kidney transplantation upper ureteral strictures and former kidney tuberculosis [1–8]. Although this procedure is not routinely performed due to its specific indications, it has been applied both in the adult and pediatric populations [4, 9].

Nowadays, due to the evolution of minimally-invasive surgery, there is a shift in the scientific interest of surgeons from open surgery to laparoscopic and robotic-assisted laparoscopic surgery [10, 11]. The latter approach provides unique characteristics, such as enhanced precision and control in movements with a motion scaling feature, a greater range of motion with seven degrees of freedom, 3D high-resolution visualization and ergonomic design of the robotic platform [12]. For these specific features, the robotic-assisted laparoscopic ureterocalicostomy (RALUC) is gaining ground, ultimately, since it provides delicate dissection and suturing of vital tissues. The current systematic review summarizes all applications of RALUC in both adults and children.

## Patients and methods

### Search strategy

Following the establishment of an a priori protocol (registered to https://osf.io on 25 January 2025, DOI: 10.17605/OSF.IO/YCQN6), and in line with the Preferred Reporting Items for Systematic Reviews and Meta-Analyses (PRISMA) Guidelines, three different databases (PubMed, Scopus and Cochrane) were screened, from their inception up to 16 February 2025. The search was limited to studies involving humans and articles published in English. Manual search was also performed, while additional potentially relevant studies were identified, carefully screening the references of included studies. Both peer-reviewed journal articles and abstracts from major congresses (EAU, WCE, AUA, SIU) were considered eligible. The following search string was used: robotic AND (ureterocalicostomy OR ureterocalicostomies OR ureterocalycostomy OR ureterocalycostomies) .

### Selection criteria and data extraction

The PICO (Patients, Intervention, Comparison, Outcome) framework was used for screening the databases. Eligible patients (P) should be adults (>18 years-old) and children of all ages with UPJO and complex renal anatomy or failed previous pyeloplasty or with proximal ureteral strictures. The intervention (I) should be RALUC. Included studies should compare (C) RALUC with conventional open or laparoscopic ureterocalicostomy and with other approaches, such as pyeloplasty. Nevertheless, articles investigating the feasibility, the safety and the efficacy of RALUC in general, without comparison groups were also included. The primary outcomes (O) were surgical time, success rates and complication rates, according to the Clavien-Dindo Classification System [13]. The secondary endpoints were hospitalization time, conversion rates, estimated blood loss and reoperation rates. Included studies were prospective ones (randomized, quasi-randomized or non-randomized) comparing RALUC with conventional open or laparoscopic ureterocalicostomy and with other approaches such as pyeloplasty, as well as retrospective comparative ones. However, non-comparative studies and case-series investigating the feasibility, the safety and the efficacy of RALUC, in general, were also accepted. Studies including different approaches were eligible only if they provided separate data for RALUC. Patients who had undergone previous interventions, such as pyeloplasty (open, laparoscopic or robotic), were included.

### Article selection

**Fig. 1:**
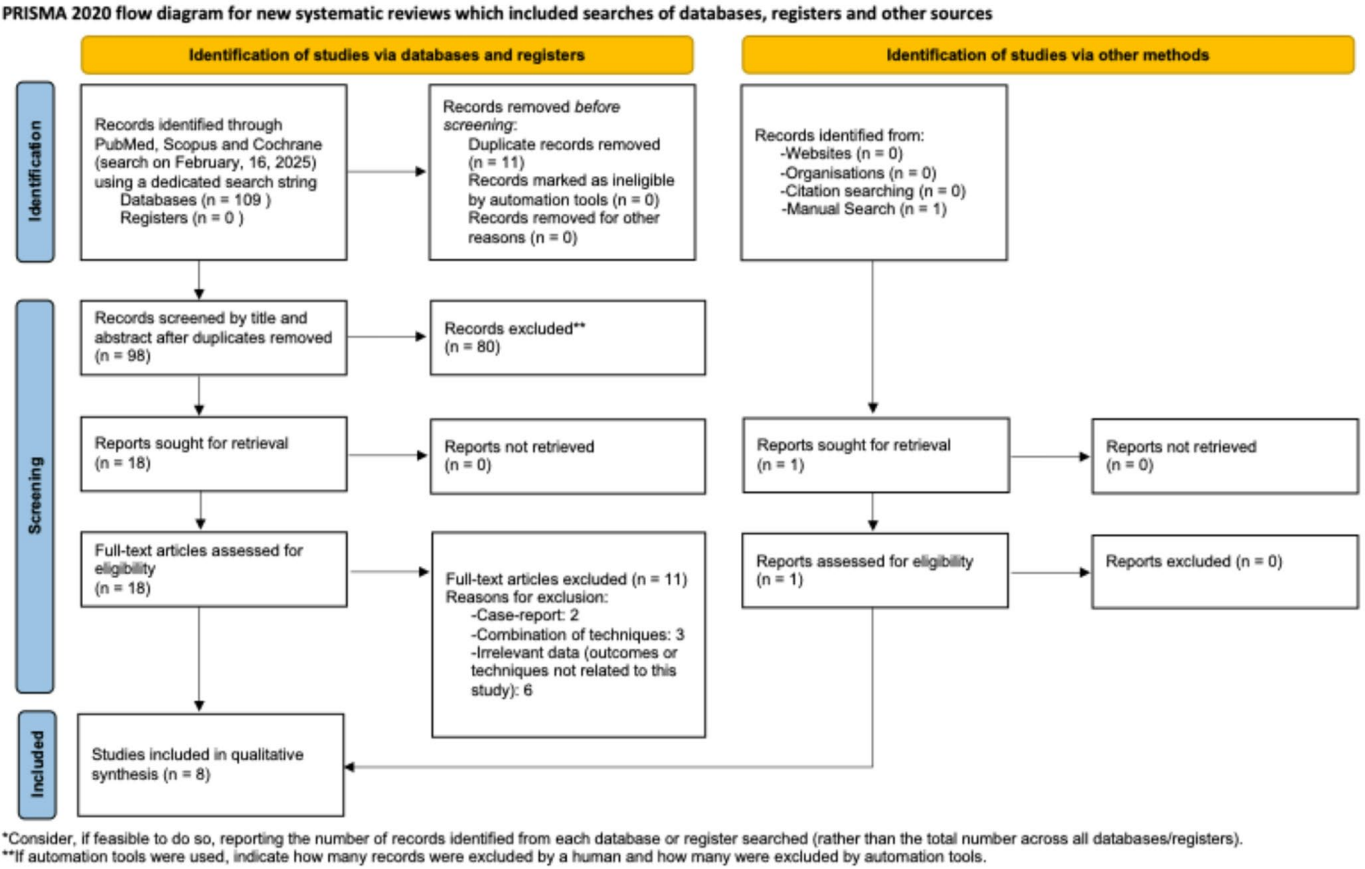
The PRISMA Flowchart, showing the selection of included studies

The three databases were independently screened by two of the authors (T.S. and P.K.), taking into consideration both the inclusion and exclusion criteria. Potential disagreements between the two authors were resolved by a third independent author (J.U.S.) until an agreement was made. 109 articles were found during the primary search (PubMed: 31, Scopus: 78, Cochrane: 0). When 11 duplicates were removed, 98 studies were investigated by carefully reading the title and the abstract. Of these, 80 articles were excluded and 18 full-text articles were evaluated for eligibility. Using the SQR3 method (Survey, Question, Read, Recite, and Review), seven studies were selected for qualitative analysis, while 11 were excluded. Two of the excluded studies were case-reports, three studies reported the use of a combination of techniques and six studies reported outcomes and techniques that were unrelated to this systematic review. One study was added to the final qualitative synthesis after a manual search. Figure 1 illustrates the PRISMA Flow chart for the study selection process.

## Results

### Studies characteristics

The final qualitative synthesis included eight studies, which fulfilled all inclusion criteria [14–21]. Table 1 summarizes all characteristics of included studies. The oldest article was published in 2008 by Casale et al. [19], while the most recent one was published in 2025 by Stolzenburg et al. [21]. The number of included patients ranged from four [16] to 24 [20], reflecting the rare nature of the implementation of RALUC. Four studies reported the outcomes of RALUC in the adult setting [14, 17, 18, 21], while four studies were pediatric ones [15, 16, 19, 20]. Although in most studies, the robotic system that was used was not defined [15, 16, 18–20], the robotic platform which was utilized ranged among the remaining systems [14, 17, 21]. Almost all studies were case-series [14, 16–21], while one study was defined as retrospective by the authors, even though it included a small sample size [15]. Likewise, almost all studies were non-comparative ones [14, 16–21], except one, which compared robotic and laparoscopic ureterocalicostomy with laparoscopic and robotic pyeloplasty [15]. This particular study included both laparoscopic and robotic procedures in the ureterocalicostomy cohort. Although data were not reported separately for the two approaches, this study was included in the systematic review, because minimally-invasive ureterocalicostomies are a very rare sample. Finally, the level of evidence, according to Oxford Centre for Evidence-Based Medicine [22] was 4 in seven studies [14, 16–21] and 2b in one study [15].

**Table 1:** Characteristics of included studies

Study Name	Journal/Year of Publication	Number of Patients	Population	Robotic Platform	Type of Study	Comparison Group	Level of Evidence*
Ramanitharan et al. [14]	Journal of Laparo-endoscopic & Advanced Surgical Techniques/ 2019	6	Adults	Da Vinci Xi system with NIRF technology	Case-series	No	4
Esposito et al.[15]	International Urology and Nephrology/ 2022	6	Pediatric	N/A	Retro-spective	Yes, with laparoscopic and robotic pyeloplasty	2b
Adamic et al. [16]	BJUI Compass/ 2021	4	Pediatric	N/A	Case-series	No	4
Xu et al. [17]	The Canadian Journal of Urology/ 2024	6	Adults	Da Vinci Single Port (SP) Platform	Case-series	No	4
Chhabra et al. [18]	Arab Journal of Urology/ 2016	5 (6 renal units)	Adults	N/A	Case-series	No	4
Casale et al. [19]	The Journal of Urology/ 2008	9	Pediatric	N/A	Case-series	No	4
Mittal et al. [20]	The Journal of Urology/ 2022	24	Pediatric	N/A	Case-series	No	4
Stolzenburg et al. [21]	Hellenic Urology/ 2025	9	Adults	X or Xi Da Vinci robotic system	Case-series	No	4

*According to Oxford Centre for Evidence-Based Medicine: Levels of Evidence (March 2009), https://www.cebm.ox.ac.uk/resources/levels-of-evidence/oxford-centre-for-evidence-based-medicine-levels-of-evidence-march-2009

### Patients baseline characteristics

The preoperative data of included studies are presented in Table 2. Patient age ranged from 5.1 (IQR: 1.9–14.7] [20] to 58.78 ± 12.03 [21] years-old, while patient body mass index (BMI) ranged from 18.63 (range 14.46–22.2) [16] to 22.7 (range 18.0–48.0) [17]. The male-to-female ratio ranged from 1/2 [14, 16, 17] to 11/4 [15]. As far as the side of ureterocalicostomy is concerned, ,a slight predominance for the right side has been reported in included studies [14–21]. Regarding the etiology of ureterocalicostomy, the most frequently reported one was previous failed pyeloplasty, while other indications included intrarenal pelvis, malrotation, previous pyelolithotomy, previous partial nephrectomy, previous percutaneous nephrolithotomy (PCNL), exaggerated intrarenal collecting system, short ureter and extensive scarring at the ureteropelvic junction [14–21]. The length of the stricture was reported in only one study, ranging from 1 to 3 cm [14]. Most patients were symptomatic before the operation, while frequently reported symptoms included flank or abdominal pain, fever, urinary tract infections (UTIs) and anuria [14–21]. Finally, the rate of patients who had undergone previous pyeloplasty ranged in included studies from 20% [18] to 100% [14, 16], while the rate of patients who had undergone a nephrostomy tube placement before the ureterocalicostomy ranged from 38% [20] to 100% [14, 18].

**Table 2:** Preoperative characteristics of included Studies

Study Name	Age (years-old)	BMI (kg/ m^2^)	Male/ Female	Side	Etiology for RALUC	Length of Stricture	Presence of Symptoms	Prior Pyelo-plasty	Nephro-stomy Tube*
Ramanitharan et al. (mean) [14]	33.7 (18–41)	N/A	1/2	Right *n* = 2 (33.3%), left *n* = 4 (66.6%)	Previously failed pyeloplasty *n* = 6 (100%)	1–3 cm range	Flank pain *n* = 6 (100%), fever and pyone-phrosis *n* = 1 (16.6%)	*n* = 6 (100%)	*n* = 6 (100%)
Esposito et al. (median) [15]	10.1 (range 3–17)	41.1 weight (kg) (range 15–70)	11/4	Right *n* = 9 (60%), left *n* = 6 (40%)	Intrarenal pelvis *n* = 5 (33.3%), malrotation *n* = 2 13.3%), failed pyeloplasty *n* = 8 (53.3%)	N/A	All *n* = 14 (93.3%), flank/ abdominal pain *n* = 7 (46.7%), UTIs *n* = 4 (26.6%), pain and UTIs *n* = 3 (20%)	*n* = 8 (53.3%)	N/A
Adamic et al. (mean) [16]	11 months to 14 years-old (range)	18.63 (range 14.46–22.2)	1/2	N/A	Prior failed pyeloplasty *n* = 4 (100%)	N/A	Anuria (solitary kidney) *n* = 1 (25%), flank pain *n* = 1 (25%)	*n* = 4 (100%)	*n* = 3 (75%)
Xu et al. (median) [17]	47 (range 19–68)	22.7 (range 18.0–48.0)	1/2	Right *n* = 6 (50%), left *n* = 6 (50%)	N/A	N/A	N/A	*n* = 2 (33.3%)	*n* = 5 (83.3%)
Chhabra et al. (median) [18]	33.7 (18–41)	N/A	2/3	Right *n* = 4 (80%), bilateral *n* = 1 (20%)	Previous pyelolithotomy *n* = 3 (60%), previous PCNL *n* = 1 (20%), previous failed pyeloplasty *n* = 1 (20%)	N/A	Flank pain *n* = 5 (100%), UTI *n* = 1 (25%), obstructive uropathy (solitary kidney) *n* = 1 (20%)	*n* = 1 (20%)	*n* = 5 (100%)
Casale et al. (mean) [19]	6.5 (range 3–15)	N/A	N/A	Right *n* = 4 (44.4%), left *n* = 5 (55.5%)	Recurrent obstruction after primary pyeloplasty *n* = 6 (66.6%), exaggerated intrarenal collecting system *n* = 3 (33.3%)	N/A	N/A	*n* = 6 (66.6%)	N/A
Mittal et al. (median) [20]	5.1 (IQR: 1.9–14.7)	N/A	17/7	Right *n* = 14 (58%), left *n* = 10 (42%)	Short ureter *n* = 3 (13%), intrarenal pelvis *n* = 5 (21%), extensive scarring at the ureteropelvic junction *n* = 16 (67%)	N/A	Pain *n* = 2 (10%), UTI *n* = 4 (19%)	*n* = 21 (86%)	*n* = 9 (38%)
Stolzenburg et al. [21] (mean)	58.78 ± 12.03	N/A	N/A	Right *n* = 4 (44.4%), left *n* = 5 (55.5%)	Previous failed pyeloplasty *n* = 3 (33.3%), previous stone surgery *n* = 2 (22.2%), previous partial nephrectomy *n* = 1 (11.1%), intrarenal pelvis *n* = 3 (33.3%)	N/A	N/A	*n* = 3 (33.3%)	N/A

*BMI* body mass index (kg/m^2^), *RALUC* Robotic-Assisted laparoscopic Ureterocalicostomy, *UTI* urinary tract Infection, *PCNL* percutaneous Nephrolithotomy, *IQR* interquartile range

*Prior to surgery

### Intraoperative and postoperative characteristics

The intraoperative and postoperative parameters of included studies are summarized in Table 3. Total operative time ranged from 157.6 (90–240) [15] to 272 (210–356) [20] minutes, while mean console time was reported only in one study, being 135 min [14]. Estimated blood loss (EBL) ranged from 27.5 (range 10–75) [16] to 115 (50–200) mL [14], while hospital stay ranged from 21 (range 17–26) [19] hours to 6.5 (5–8) days [18]. The JJ stent removal time ranged from 4 weeks [14, 17, 18] to 74 (36-95.5) days [8]. Reoperation rates ranged from 0% [14, 15, 19, 21] to 50% [17], while the success rates ranged from 66.7% [17] to 100% [14–16, 19]. Finally, taking into consideration the Clavien-Dindo Classification System [13], the Grade I-II complications ranged from 0% [16, 17, 19, 21] to 40% [18], while the Grade III-IV ones ranged from 0% [14–16, 19, 20] to 20% [18]. A detailed list of complications of included studies is presented in Supplementary Table 1.

**Table 3:** Intraoperative and postoperative characteristics of included Studies

Study Name	Total Operative Time (min)	Console Time (min)	Estimated Blood Loss (mL)	Hospital Stay (days)	JJ Time (days/ weeks)	Reope-ration	Success Rates	Grade I-II Compli-cations*	Grade III-IV Compli-cations*
Ramanitharan et al. (mean) [14]	178 (range 140–240)	135	115 (50–200)	6.1 (5–8)	4 weeks (all patients)	*n* = 0 (0%)	100% median follow-up 15 (6–22) months)	*n* = 2 (33.3%)	*n* = 0 (0%)
Esposito et al. (median) [15]	157.6 (90–240)	N/A	N/A	2.8 (2–10)	4–6 weeks	*n* = 0 (0%)	100% median follow-up 37.2 (6–60 months)	*n* = 3 (20%)	*n* = 0 (0%)
Adamic et al. (mean) [16]	208	N/A	27.5 (range 10–75)	3.5	4–6 weeks	*n* = 1 (25%) nephre-ctomy (unreal-ted to surgery)	100% mean follow-up 4.46 years	*n* = 0 (0%)	*n* = 0 (0%)
Xu et al. (median) [17]	248.5 (172–324)	N/A	N/A	1 (0–2)	4 (2–12) weeks	*n* = 3 (50%) (2 endosco-pic balloon dilations, 1 diagno-stic URS	66.7% median follow-up 10.35 months (1.1–51.6)	*n* = 0 (0%)	*n* = 1 (16.6%)
Chhabra et al. (median) [18]	172 (144–260)	N/A	100 (50–250)	6.5 (5–8)	4 weeks all patients	*N* = 1 (20%) balloon dilatation and re-stenting	83.3% median follow-up 11 (7–48) months	*n* = 2 (40%)	*n* = 1 (20%)
Casale et al. (mean) [19]	168 (102–204)	N/A	N/A	21 h (range 17–26)	6 weeks all patients	*n* = 0 (0%)	100% at 6 and 12 months postope-ratively	*n* = 0 (0%)	*n* = 0 (0%)
Mittal et al. (median) [20]	272 (210–356)	N/A	N/A	2 (1–2)	74 days (36-95.5)	*n* = 2 (8%) endo-scopic interve-ntions and both ultimately underwent nephrecto-my	92% median follow-up 16.1 months (IQR: 6, 47.5)	*n* = 3 (13%)	*n* = 0 (0%)
Stolzenburg et al. [21] (mean/ median)	184.78 ± 53.35 (mean)	N/A	50.00 (17.50–110.00) (median)	N/A	40.00 days (25.00–67.00)	*n* = 0 (0%)	88.8%	*n* = 1 (11.1%)	*n* = 1 (11.1%)

*JJ* Double-J stent, *URS* Ureteroscopy, *IQR* interquartile range

*According to Clavien-Dindo Classification System

## Discussion

### History of Minimally-Invasive ureterocalicostomies

The ureterocalicostomy procedure was first described, as an open approach, by Neuwirt et al. in 1947 for the management of complicated cases of UPJO syndrome [23]. The first reference of laparoscopic ureterocalicostomy in the literature was reported by Gill et al. in a series of two patients in 2004 [23]. The procedure was successful in one patient, but the second patient underwent nephrectomy later on, despite an initial radiological improvement [24]. Finally, the first RALUC procedure was reported by Korets et al. in 2007 [25]. However, the authors described using a hybrid technique, including laparoscopic maneuvers for the dissection part and then they enabled the robotic instruments for performing the reconstruction [25]. The first “totally robotic” RALUC procedure was later reported by Schimpf et al. in 2009 in a 32 years-old female patient, while the authors reported the long-term outcomes of this procedure. Interestingly, this woman required a nephrostomy tube placement two years after the procedure, during the third trimester of her pregnancy, due to flank pain [26]. Nevertheless, after labor, the nephrostomy tube was removed, following a normal nephrostogram [26].

### RALUC in the adult setting

Chhabra et al. reported in their case-series the outcomes of six RALUC procedures that were performed in their Center from 2011 to 2015 in five patients (one patient underwent bilateral RALUC) [18]. Peculiarities of their technique included the placement of an open-ended pigtail ureteric catheter over a guidewire before the placement of the trocars and use of ultrasound intraoperatively for detecting the lower pole calyces. All patients in this cohort had secondary UPJO due to previous pyelolithotomy, percutaneous nephrolithotomy (PCNL) or failed pyeloplasty procedures. The authors reported no conversions to laparoscopic or open surgery, while two patients presented with Grade I and one patient with Grade IIIb complications according to Clavien-Dindo postoperatively [13]. Five out of six procedures were successful during the follow-up period [18]. Ramanitharan et al. took advantage of the Near-Infrared Fluorescence Imaging (NIRF) technology of the Da Vinci Xi robotic system and reported their outcomes of six RALUC procedures which were performed with this approach [14]. All patients had secondary UPJO due to a previous failed pyeloplasty. The authors reported activating the NIRF mode, after injecting 2.5 mL of indocyanine green (ICG) intravenously, after disconnecting the lower pole of the kidney from the adjacent perinephric fat and disconnecting the stenotic segment of the ureter and before performing the partial nephrectomy of the lower pole. With the aid of the NIRF technology, the vasculature of the distal part of the dissected ureter was assessed, minimizing the restricture rates. The next steps were spatulation of the ureter and implementation of the anastomosis. Two patients developed Grade I-II complications according to Clavien-Dindo, while no recurrences were reported during the follow-up period. However, one patientshowed persistent hydronephrosis and was under close observation up to the time of the publication [14].

Xu et al. reported their experience with the Da Vinci SP system on ureterocalicostomies [17]. In total, six patients underwent RALUC from 2020 to 2023, four of whom have undergone previous surgical interventions, including pyeloplasty, balloon dilation and pyeloplasty. Interestingly, two patients had simultaneous proximal strictures, along with the UPJO, requiring concurrent reimplantation. The configuration of their instruments was the following: an SP endoscope at six o’clock, a monopolar curved scissors at nine o’clock and a Maryland bipolar at none o’clock (three and nine o’clock were switched depending on the laterality of the lesion). One Grade IIIb complication was reported, according to Clavien-Dindo, while five out of six procedures were proven to be successful during the follow-up period [17]. Finally, Stolzenburg et al. reported their outcomes of nine adult patients undergoing RALUC in two German Centers [21]. The authors described a standardized technique for RALUC and provided some tips and tricks. In their case-series, the postoperative course was uneventful for seven patients, while one patient needed a prolonged JJ stent due to extravasation and one patient needed nephrostomy tube placement, due to notable hydronephrosis. This last patient finally had a stricture recurrence and complete obstruction was evidenced [21].

### RALUC in pediatric patients

Mittal et al. presented one of the largest RALUC series performed in children [20]. In their study, the outcomes of 24 procedures were analyzed, 86% of which had undergone previous pyeloplasty surgery. The indications for performing RALUC in their series were short ureter, scarring of the ureteropelvic junction and intrarenal pelvis. Three patients developed Grade I-II complications according to Clavien-Dindo, while no Grade III-IV ones were reported. The success rate was calculated at 92% during the follow-up period, while two patients underwent additional endoscopic procedures and both ended up with nephrectomy [20]. Likewise, Casale et al. reported their outcomes arising from nine consecutive pediatric patients who underwent RALUC [19]. Six patients had previous failed pyeloplasty procedures, while three patients had exaggerated intrarenal systems. Interestingly, the authors reported applying running sutures for the posterior anastomosis and interrupted sutures for the anterior anastomosis, which were then tied in groups. The authors reported zero complication rates and 100% success rates at 6-months and 12-months postoperatively [19]. With some words of wisdom in their discussion section, the authors declared that recurrence of the obstruction is the most feared complication of the procedure, while this complication can be avoided by a generous excision of the renal parenchyma during partial nephrectomy and by performing a tension-free anastomosis, avoiding ischemia and scar tissue formation. In that effort, JJ catheters can eliminate urine leakage, which promotes scar tissue development [19].

Adamic et al. reported some practical tips and technical modifications for RALUC in children [16]. All children in their case-series have undergone previous pyeloplasty procedures, while no complications were reported. Although all cases were anatomically successful, one patient finally needed nephrectomy due to inadequate drainage of the collecting system, despite a broad intrarenal space. Their tips included taking advantage of flexible nephroscopy for detecting the dependent calyces, use of harmonic scalpel for performing the nephrotomy, so as to avoid bleeding and clamping and implementation of stay sutures or hitch stitches for the reconstruction due to the mobility of the renal lower pole. Moreover, they suggested pre-placing all anastomotic sutures to the calyces, because they are fragile and tear easily, and placement of drainage in all cases in an effort to avoid urine leakage [16]. Finally, Esposito et al. also reported their experience with RALUC in children [15]. Although the authors reported performing both laparoscopic (nine cases) and robotic (six cases) ureterocalicostomies, this study was included in the systematic review, because the authors reported using the same surgical steps in both approaches and due to the rare nature of minimally-invasive ureterocalicostomies. This particular study was actually a comparative retrospective one, comparing minimally-invasive ureterocalicostomy with minimally-invasive Anderson-Hynes pyeloplasty. The authors reported that minimally-invasive ureterocalicostomy is a safe and efficient alternative approach for UPJO repair in both primary and salvage settings. Interestingly, the authors reported that the robotic approach was the preferred option for treating recurrent UPJO in both groups [15]. Interestingly, as shown in **Supplementary Table 2**, the success rates were higher and the complication rates were lower in the pediatric series, compared with the adult ones.

### Ureterocalicostomy indications

Several indications for performing ureterocalicostomy can be found in the literature, including previous failed pyeloplasty procedures and UPJO which is associated with anatomical abnormalities, such as renal fusion, rotation and ascent or intrarenal pelvis and inadequate ureteral length [27]. Moreover, ureterocalicostomy can be performed for the management of certain patients with proximal ureteral strictures [28]. In our systematic review, the most frequently reported indication was previous failed pyeloplasty, followed by UPJO with intrarenal or inadequate pelvicalyceal system space. Other indications, reported in included studies, were malrotation, previous pyelolithotomy or PCNL procedures, exaggerated intrarenal collecting system, previous partial nephrectomy, short ureter and extensive scarring at the ureteropelvic junction [14–21].

### Critical appraisal of robotic ureterocalicostomy outcomes

Total operative time surpassed two hours in all studies, ranging from 157.6 (90–240) [15] to 272 (210–356) [20] minutes. Success rates were generally high ranging from 66.7% [17] to 100% [14–16, 19], while reoperation rates ranged from 0% [14, 15, 19, 21] to 50% [17]. Regarding the complications, these ranged from 0% [16, 19] to 60% [18] in included studies, with the most serious being the necessity of nephrostomy tube placement, reoperation and nephrectomy [14–21]. These outcomes are in line with the outcomes of open ureterocalicostomy series in both adults and children [4, 9, 29, 30], with only EBL and hospital stay being beneficial for the robotic series. Although the majority of these data arise from low-quality case-series and thus generalization of results must be performed with caution, these findings underline the well-established dogma that the operative technique is more important than the approach (open versus minimally-invasive) that is used.

### Limitations

This systematic review is not without limitations. An important limitation is that only eight studies were included. However, RALUC is a rarely performed procedure, while many studies reported a combination of techniques without providing separate data for robotic ureterocalicostomies. Moreover, data were highly heterogeneous and consequently a meta-analysis could not be performed. A quantitative synthesis on operative time or success rates was also not feasible due to the non-manageable heterogeneity, with different research questions, indications, surgical techniques, robotic platforms and populations included, while several studies reported a combination of techniques or outcomes. Although comparative studies were eligible in the PICO framework that was used, most included studies were case-series, except one study which was a retrospective comparative one. A risk of bias assessment was not possible, due to high heterogeneity and low quality of included studies, including exclusively case-series and retrospective ones. We thus used alternatively the levels of evidence, according to the Oxford Centre for Evidence-Based Medicine [22]. Finally, all procedures were performed by highly experienced surgeons and in large-volume centers. It is thus possible, that some beneficial outcomes reported in this systematic review may not be reproduced in less experienced hands.

## Conclusions

RALUC is a feasible, safe and efficient procedure for patients with complicated UPJO, especially in cases with previous failed pyeloplasty or with intrarenal pelvis and other renal anomalies. Both adults and pediatric patients can take advantage of the minimally-invasive procedures, while technological advancements of the robotic systems, such as NIRF technology and SP platforms may prove especially helpful. The implementation of higher-quality studies on larger samples, including comparative ones and randomized controlled trials (RCTs) is crucial, in order to draw safer conclusions.

## Supplementary Information

Below is the link to the electronic supplementary material.

Supplementary Material 1

Supplementary Material 2

## Data Availability

No datasets were generated or analysed during the current study.

## Abbreviations

RALUC: Robotic-assisted laparoscopic ureterocalicostomy
PRISMA: Preferred Reporting Items for Systematic Reviews and Meta-Analyses
UPJO: Ureteropelvic junction obstruction
PICO: Patients, Intervention, Comparison, Outcome
SQR3: Survey, Question, Read, Recite, and Review
BMI: Body mass index
PCNL: Percutaneous nephrolithotomy
UTIs: Urinary tract infections
EBL: Estimated Blood Loss
NIRF: Near-Infrared Fluorescence Imaging
ICG: Indocyanine green
