# Supplementary material for: Robotic-assisted laparoscopic ureterocalicostomy (RALUC): a systematic review of its applications
Source: World J Urol. 2025 Dec 17;44(1):47. doi: 10.1007/s00345-025-06046-w (PMC12712003; doi:10.1007/s00345-025-06046-w)
Supplement: Supplementary file 1 — Supplementary Material 1 [file 345_2025_6046_MOESM1_ESM.docx]

| **Study Name** | **Grade**  **I-II Complications** | **Grade**  **III-IV Complications** |
| --- | --- | --- |
| **Ramanitharan et al. (mean)^14^** | n=2 (fever and UTIs) | n=0 |
| **Esposito et al. (median)^15^** | n=3 | n=0 |
| **Adamic et al. (mean)^16^** | n=0 | n=0 |
| **Xu et al. (median)^17^** | n=0 | n=1 (nephrostomy tube placement due to urine leak) |
| **Chhabra et al. (median)^18^** | n=2 (fever) | n=1 (balloon dilatation and re-stenting) |
| **Casale et al. (mean)^19^** | n=0 | n=0 |
| **Mittal et al. (median)^20^** | n=3 | n=0 |
| **Stolzenburg et al.^21^ (mean/ median)** | n=1 (prolonged JJ stent) | n=1 (nephrostomy tube placement, due to notable hydronephrosis) |

**Supplementary Table 1:** A detailed list of Complications which were reported in included studies.
