# Supplementary material for: Robotic-assisted laparoscopic ureterocalicostomy (RALUC): a systematic review of its applications
Source: World J Urol. 2025 Dec 17;44(1):47. doi: 10.1007/s00345-025-06046-w (PMC12712003; doi:10.1007/s00345-025-06046-w)
Supplement: Supplementary file 2 — Supplementary Material 2 [file 345_2025_6046_MOESM2_ESM.docx]

| **Population** | **Study Name** | **Success Rates** | **Complication Rates** |
| --- | --- | --- | --- |
| Adults | **Ramanitharan et al.^14^** | 100% | 33.3% |
|  | **Xu et al.^17^** | 66.7% | 16.6% |
|  | **Chhabra et al.^18^** | 83.3% | 60% |
|  | **Stolzenburg et al.^21^** | 88.8% | 22.2% |
| Pediatric | **Esposito et al.^15^** | 100% | 20% |
|  | **Adamic et al.^16^** | 100% | 0% |
|  | **Casale et al.^19^** | 100% | 0% |
|  | **Mittal et al.^20^** | 92% | 13% |

**Supplementary Table 2: Analysis of Adult versus Pediatric Outcomes**
